# Application of Human-Computer Interaction System in Regional Tourism Competitiveness Analysis under IoT Background

**DOI:** 10.1155/2022/3849610

**Published:** 2022-09-22

**Authors:** Zhenli Jia, Jing Zhang, Zhongquan Cui

**Affiliations:** ^1^Faculty of Business, Yuxi Normal University, Yuxi 653100, China; ^2^Faculty of Business, City University of Macau, Macau 999078, China

## Abstract

With the continuous development of Internet of Things technology, this technology is not limited to the laboratory but is more and more applied in the process of commercialization. Large-scale machine communication is one of the three most important scenarios for 5G applications. Its features include larger uplink access, smaller data packets, higher device energy consumption requirements, lower device transmission speed, higher latency, and bursty communication. With the development of the Internet of Things in the 5G era, interpersonal interaction experience has been further optimized and welcomed by the market and adopt Qt/embedded multilayer menu design mode to improve our computer system interaction to facilitate the operation and Maintenance system. The software function of the human-computer interaction system has reached the expected result. By constructing the application of a human-computer interaction system in the analysis of regional tourism competitiveness, we analyze the temporal and spatial changes of tourism competitiveness in four major economic regions in China and combine multiple linear regression and geographic detector models to analyze China's tourism competitiveness. On this basis, we propose to further enhance the tourism competitiveness of areas with weak tourism development infrastructure and encourage potential tourism development areas to complement each other with high-quality resources between regions.

## 1. Introduction

The development of the Internet of Things has promoted the progress of all aspects of the city, from the big smart city to the small smart home, all continue to develop [1]. In this process of development, there are various demands, so the network must be connected quickly with the actual scenes of different fields, so as to promote the optimization and development of all aspects and achieve the goal of multiaspect connection [2]. In general, 5G provides a good network infrastructure and technical support for Internet applications, as well as communication between people and communication between people (MTC), which allows the network to be more extensively used in the industry [3]. Because we are in such a big era, we use the Windows CE platform of SQLite database to develop a human-computer interaction system and establish a reasonable local storage structure [4]. Compared with the old version of the database system, the SQLite database is usually smaller, portable, easy to use, and has more complete functions to complete data management tasks and perform fast and effective search queries to meet the visual human-machine interface (HMI) system [5]. The product's requirements for database management will further create huge value. Because the menu components, graphic icons, and language information of the oscilloscope interactive interface maintain the same height, the same size, and the unified sequence, the oscilloscope has attracted the attention of many manufacturers, and it is this factor that speeds up the oscilloscope's man-machine Development and design of interactive systems. Through the interactive experience provided by the computer interactive system, oscilloscope manufacturers can attract more social users, further increase the loyalty of oscilloscope users, and significantly increase the value of oscilloscope products [6]. The competition in the regional tourism industry has become increasingly fierce as the people's material needs continue to increase [7]. It is also because of this factor that the tourism industry is at the forefront of the development of the national economy, and it has also stimulated the strategic position of the tourism industry in China [8]. With the support of local governments, competition in the region is becoming increasingly fierce. Cities, autonomous regions, and municipalities have placed tourism development in an important strategic position, making regional tourism a key industry and core industry.

## 2. Related works

Some research uses Qt/Embedded multi-layer menu setting mode to improve the human-computer interaction of the system and makes it easier for employees to operate and maintain the system [9]. Because the SQLite database file is small, it can save space. The software functions of the human-computer interaction system can achieve the predetermined design goals [10]. Some research through the research and development of various products, related tourism service departments, administrative departments, and local tourism companies in the region to improve the infrastructure, so that the region can meet the needs of tourists [11]. In turn, economic and social benefits can be obtained. Some research under the background of the increasing influence of information on tourism competition has conducted research on the query behavior of tourism information, established a theoretical research model, and provided information basis for tourists [12]. And it can be used to query usage methods and influencing factors. Some research based on the Qt platform centered on the oscilloscope human-computer interaction interface, systematically designed the oscilloscope human-computer interaction, and designed a custom font library that satisfies the interface display requirements to achieve mutual response [13]. Some research shows a reliable transmission method based on NOMA without the need for distributed scheduling. Since the random access mechanism of nondistributed transmission eliminates the risk of instability, we first studied the stability of the distributed free reservation access system based on the Foster–Lyapunov standard fast retransmission mechanism and then provided a stable noncooperative system to Control the probability of distributed transmission [14].

## 3. Human-Computer Interaction System Design for 5g Internet of Things

### 3.1. 5G Internet of Things Dispatch-free NOMA Model Design

**Figure 1:**
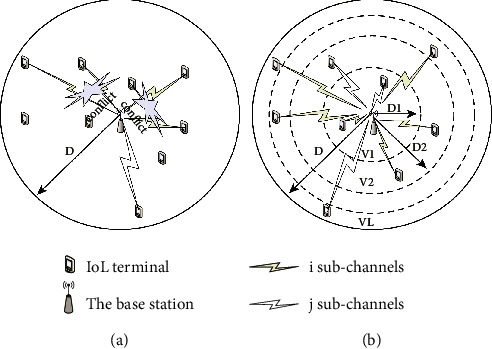
Represents the schematic diagram of unplanned transmission.

NOMA technology increases the power resources of the power domain. Therefore, the machine uses CO (connection option) instead of blocking the source at regular intervals to display the connection source. The number of connection options at any given time in the OMA system can only be controlled by the number of available low access points, which is determined by the number of connection options. Due to the limited resources of the OMA system, the available connection options are not sufficient for large-scale transmission whenever there is available space, as shown in Figure 1(a). At the same time, in the case of the uplink transmission of the communication channel of the base station, considerable competition occurs at the receiving end. The schematic diagram of the distributed hierarchical NOMA strategy is shown in Figure 1(b).

First, set the received power level and the corresponding received power in advance. For example, there are *L* predetermined target received powers within the range of the base station, and the received power *V* is very large, from large to small to >.,>...>*v*. If the SNR of the receiving end must be at least *T*, and the decoding order of the receiving end depends on the magnitude of the received power, it includes the interference of the same frequency of the *L* signal and the “interference” of the noise and are

(1) $$\begin{array}{c} V_{1}=\left\{\begin{array}{cc} \overset{L}{\underset{k=1+1}{\sum }}v_{k}\text{,} & l=1,\ldots ,L-1\text{,}\\ 0\text{,} & l=L\text{.} \end{array}\right. \end{array}$$

We can obtain *v* = *T*(*T* + 1) based on *v* = *F*(V + 1)*v* = *T*(*T* + 1). The received power with the smallest received power difference can also be obtained, which helps increase the value of L. In practical application scenarios, it can be considered that the farther away from the base station, the greater the signal fading caused by large-scale fading. At this time, in order to reduce the transmission power of device_uplink communication, according to the distance from the base station, the entire cell is divided into *L* circular areas called *L* layers. Taking into account the uniform distribution of equipment, the standard for dividing layers is divided according to the area of the following areas, namely,

(2) $$\begin{array}{c} D_{l}=\left\{\begin{array}{cc} D\sqrt{\frac{l}{L}}\text{,} & l=1,\ldots ,L\text{,}\\ 0\text{,} & l=0\text{.} \end{array}\right. \end{array}$$

According to the distance from short distance to long distance, the estimated reach power of each device layer is v. Devices on the same layer have the same functions. In this way, the equipment far away from the base station can be reduced to make the power reach the expected level, and the transmission power of the equipment of the entire system can be reduced. As shown in Figure 1(b), in this mechanism, the cell is divided into *L* concentric circles, and IoT devices in different source areas have different target uplink received powers. Since the gain of other channels is considered, the more the outer loop, the smaller the target received power. The regional MTCD will determine its transmission capacity by enabling location-based segmentation and CSI. For example, for a specific MTCDk, if it is far away from the base station, the device belongs to the set *K* = {k \ *D*. <*D* ≤ *D*}, and *V* represents the standard power received. Since each MTCD has CSI information about the channel, *i* g.i- = ...M. Therefore, the transmission capacity can be calculated by calculating *P* = *v*,/maxg.k ∈ K.

Using this method, the cell is divided into *L* layers, each layer of equipment can use the resources of all channels at the same time, while the distributed layer NOMA can double the resources. The purpose is to increase the probability of successful random access and reduce signaling overhead by using unscheduled transmission to avoid the four-way handshake process of accessing resources (random access process adopted in cellular networks such as LTE).

According to the set difference rule, if there are *M* active users whose *M* ≤ *L*, the NOMA power level is set to be higher. If other power levels are selected, the *M* signal can be successfully decoded, and the receiving end is SIC to eliminate the same channel interference. However, due to the interaction with the decoding of another layer, the value of *L* cannot be too large. By selecting *M* ≤ *L* and other powers, the base station can successfully decode all signals of *M* users. Conversely, if each user selects the same power, the signal cannot be decoded.

If *M* is greater than 1, the average MTCD transmission power in the system provided by NOMA can be expressed as follows:

(3) $$\begin{array}{c} E\left[P_{k}\right]\leq \frac{\min\;\left\{2\;\ln\;2,M/M-1\right\}}{L}\overset{l}{\underset{l=1}{\sum }}\frac{v_{i}}{A_{l}}\\ =\frac{\min\;\left\{2\;\ln\;2,M/M-1\right\}}{L}\overset{L}{\underset{l=1}{\sum }}\frac{\Gamma {\left(\Gamma +1\right)}^{L-1}}{A_{0}{\left(D\sqrt{l/L}\right)}^{-\beta }}\text{.} \end{array}$$

Here, *β* represents the road loss index, and Ao represents the constant relative antenna gain.

### 3.2. Design and Analysis of Distributed Hierarchical Scheduling-Free NOMA Architecture

When MTCD selects this time slot for uplink transmission, it depends on the location, mainly based on the distance *d* from the base station. The target MTCD is at the location level 1 of the base station through *d*, the information of each MTCD channel and the information gain of the subchannel to which it belongs. According to the principle of the lowest transmission power, the power of the transmission slot is calculated as follows:

(4) $$\begin{array}{c} P_{k}=\frac{v_{l}}{\max g_{i.k}}\text{.} \end{array}$$

Here, *P* is the transmission power used by MTCD, and *v* is the power previously received by the target at the site level.

**Figure 2:**
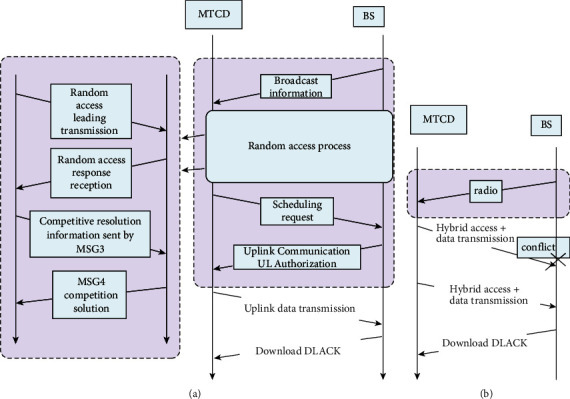
(a) Connection-oriented communication (b) Comparison of hybrid access and data transmission.

The uplink transmission uses a nonorthogonal multiplexing connection method, and the scheduling-free random access technology based on the distributed layer NOMA uses resources nonorthogonally through the signals of multiple devices on the power domain NOMA. The same frequency at the same time. It can be seen from Figure 2.

The detailed steps of the framework design are as follows:

1. Access is granted in the time slot of the cell, and each MTCD transmits its signal on the subchannel selected based on the independently calculated transmit power.
2. After the MTCD is successfully sent if the MTCD receives a DLACK signal indicating successful reception within a certain period of time, the communication is successful. Otherwise, MTCD will return to the next free time and try to connect. If the MTCD data burst is small, the signaling process of the connection-oriented method and the proposed hybrid transmission method is shown in Figure 2. For small data packets that are usually tens of bytes on the Internet of Things, the connection-oriented method will generate a large amount of signaling overhead of up to 220 bytes, and the proposed hybrid transmission method has multiple broadcast signaling overheads. Can reduce the use of bytes.

There is no separate event for power conflicts at each power level in NOMA. That is to say, if the power reaches the maximum and suddenly occurs at the power level I, it will not affect −1 level signals, even if there are a large number of levels of signals colliding, it cannot be against the power level *l* + . The signal is decoded.

In the designated distributed layer unlicensed NOMA framework, in view of the competition of C MTCD and *M* subchannels, the connection probability PCon of the first layer is expressed as follows:

(5) $$\begin{array}{c} P_{l}^{\left(im\right)}={\left(1-\frac{1}{M}\right)}^{\left(l-1\right.}{\left(1+\frac{C}{M-1}\right)}^{l-1}\text{.} \end{array}$$

Since the target of MTCD under the *K* constraint is *V*, it is first determined at the end of the receiving end, so it has nothing to do with signals of other powers, so

(6) $$\begin{array}{c} p_{c,m,1}^{smcc}=p_{c,m,1}^{sel}\text{.} \end{array}$$

The successful transmission of the MTCD signal belonging to the set *Kz* is not only related to the unique selection of the specific subchannel *m* in the hierarchical structure but also related to the MTCD selection subchannel of the set *Kr*. For *m*, the signals on this channel will not conflict. And, so

(7) $$\begin{array}{c} p_{c.m.2}^{sncc}=p_{c.m.2}^{sncc}\left\{\overset{c}{\underset{c=1}{\sum }}p_{c.m.1}^{sncc}+p_{m.l}^{nosel}\right\}\text{.} \end{array}$$

Obtained from formula (7),

(8) $$\begin{array}{c} \overset{C}{\underset{c=1}{\sum }}p_{c,m.1}^{smc}+p_{m.1}^{n_{0,c/}}=\frac{p_{c.m.2}^{sicc}}{p_{c,m.2}^{se^{\prime }}}\text{.} \end{array}$$

For any MTCD belonging to set *K*, we can obtain the following equation:

(9) $$\begin{array}{c} p_{c.m.3}^{sncc}=p_{c.m.3}^{sel}\left\{\overset{c}{\underset{c=1}{\sum }}p_{c.m.2}^{sncc}+p_{m.2}^{nosel}\left\{\overset{c}{\underset{c=1}{\sum }}p_{c.m.1}^{sncc}+pp_{m.1}^{nosel}\right\}\right\}\\ =p_{c.m.3}^{sel}\left\{\overset{c}{\underset{c=1}{\sum }}p_{c.m.2}^{sncc}+p_{m.2}^{nosel}\left\{\frac{p_{c.m.2}^{sncc}}{p_{c.m.2}^{sel}}\right\}\right\}\\ =p_{c.m.3}^{sel}p_{c.m.2}^{sncc}\left(C+\frac{p_{m.2}^{nosel}}{p_{c.m.2}^{sel}}\right)\text{.} \end{array}$$

The following rules can be found:

(10) $$\begin{array}{c} p_{c,m,l}^{succ}=p_{c,m,l}^{sel}p_{c,m,l-1}^{succ}\left(C+\frac{p_{m.l-1}^{nosel}}{p_{c,m,l-1}^{sel}}\right),l=2,3,\ldots ,L\text{.} \end{array}$$

**Figure 3:**
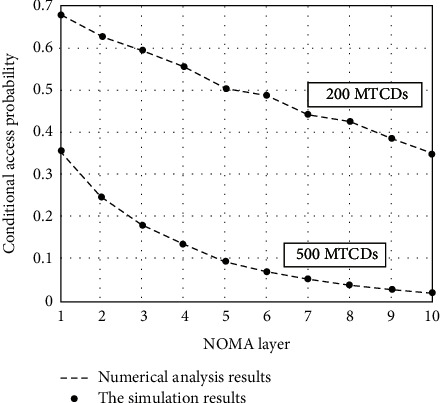
Numerical results for different presets.

Figure 3 shows the numerical value and simulation result of the connection throughput of each layer of MTCD in the formula. The greater the number of MTCDs, the lower the probability of successful connection in a single time slot that matches the actual situation. Therefore, the above-mentioned link output can accurately reflect system performance and can be used as a measure of system performance.

### 3.3. Principle of Power Layer Algorithm Selection and Joint Access Control □

Through pa = *p*(*E* + AB)·pstcc, where *p*(s/*ki*) = (∑(*j* = 1)^l ps*π*c^‘)/L, psucc^' is successfully transmitted by the MTCD of the first layer Probability. Therefore, the average transmission time of the MTCD of the system can be expressed as follows:

(11) $$\begin{array}{c} D_{the\;}=T_{P}\cdot T_{are\;}\\ =\frac{T_{P}}{P_{a}}\text{.} \end{array}$$

At present, we need to start from two aspects: firstly, to maximize the throughput of the connection, and secondly, to optimize the power limit and delay during transmission.

(12) $$\begin{array}{c} \max\;T^{Con}=\overset{L}{\underset{l=1}{\sum }}T_{l}^{Cm}\\ \;s.t.\;C1:L\in L_{T}=\left\{1.2\ldots .L_{\max}\right\}\\ C2:D_{ave}\leq D_{rc}\text{.} \end{array}$$

Among them, *C*1 and *C*2 in turn indicate that the most common constraints allowed are transmission power and average transmission delay. Lr represents the NOMA power level setting below the average transmission power limit.

Choose *L* = Lmx because the connection throughput TCon is an increasing characteristic of the NOMA power level L. P: A group that meets the average delay requirement. *P* can be obtained from the following formula:

(13) $$\begin{array}{c} P=\left\{p_{E}\;|\;\begin{array}{c} \frac{T_{p}}{p_{a}}\leq D_{req}\\ 0<p_{E}\leq 1 \end{array}\right\}\text{,} \end{array}$$

where *p*_*a*_=*p*_*E*:_/*L*_max_∑_*l*=1_^*L*^(1 − 1/*M*)^(*Q* · *p*_*E*_/*l*_max_)*l*−1^(1+*Q* · *p*_*E*_/*L*_max_/*M*−1)^∣−1^. Therefore, the optimal *p* is

(14) $$\begin{array}{c} p_{E}^{*}=arg\max_{p_{E}\in P}T^{Cnn}\text{.} \end{array}$$

If *P* is an empty set, it means that there is no optimal solution to the optimization problem even under the constraints of the maximum allowable power level and the optimal transmission control parameters. In the case that the quantity is still insufficient, *P* is determined as follows:

(15) $$\begin{array}{c} p_{E}^{*}=arg\max_{0<p_{k}\leq 1}T^{Con}\text{.} \end{array}$$

### 3.4. Human-Computer Interaction Graphical Interface Function Realization

**Figure 4:**
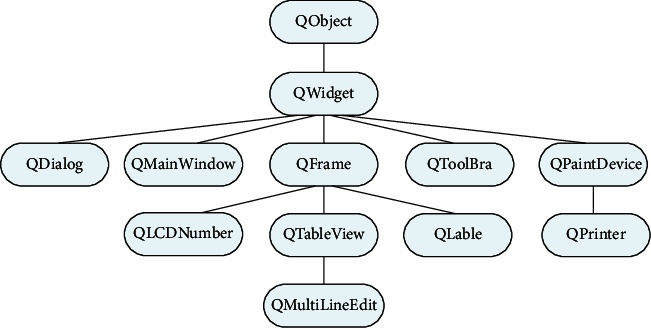
The inheritance relationship of the QWidget class.

Qt combines the functions of the digital classroom with various functions, such as buttons, input dialogs, and transfer bars. This window is an example of the QWidget class or subclass. All user window classes inherit from the QWidget class. The oldstyle relationship of the QWidget class is shown in Figure 4.

A form usually contains multiple subforms, which appear in the client area of the parent form. There are no restrictions on Qt windows. This window has top-level forms or sub-forms of other forms. When the parent form is hidden or deleted, these tasks also apply to the child form. The size and position of each part placed on the form must be appropriate.

**Figure 5:**
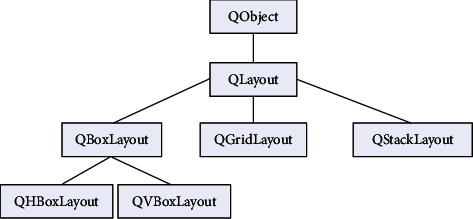
Layout manager class inheritance diagram.

These classes are usually used to improve the interaction with people and computers. The inheritance relationship of the design manager class is shown in Figure 5.

## 4. Evolution and Influencing Factors of Regional Tourism Competitiveness

### 4.1. Research Methods of Regional Tourism Competitiveness

In this study, the evolving law of China's domestic competitiveness differences and investigating the main factors affecting the competitiveness of the tourism industry are mainly entropy methods, and the coefficient of variation method is multivariate linear, including regression analysis, regional analysis, and detection. Maker and other models. Using the coefficient of variation method of the entropy method, this paper analyzes the temporal and spatial changes of the tourism competitiveness of China's four main economic regions and combines multiple linear regression analysis methods and geographic detector models to analyze the competitiveness of China's major economic regions.

The entropy method is used to determine the degree of dispersion of indicators. The calculation method and formula are as follows:

(16) $$\begin{array}{c} {r^{\prime }}_{ij}=\frac{\max\left(r_{ij}\right)-r_{ij}}{\max\left(r_{ij}\right)-\min\left(r_{ij}\right)}\text{.} \end{array}$$

Set *b*_*ij*_ = 1 + *r*_*ij*_^ ′ to make *lnf*_*ij*_ meaningful. The entropy value of each index is calculated. *H*_*ij*_ is the entropy value of the *i* indicator:

(17) $$\begin{array}{c} H_{i}=-k\overset{n}{\underset{j=1}{\sum }}f_{ij}\times \ln f_{ij}\text{.} \end{array}$$

Here, *f*_*ij*_=*b*_*ij*_/∑_*j*=1_^*n*^*b*_*ij*_, *k*=1/ln *n*(Assume *f*_*ij*_ = 0, In *f*_*ij*_ = 0). Determination of the weight of indicator *i*:

(18) $$\begin{array}{c} W_{i}=\frac{1-H_{i}}{m-\overset{m}{\underset{i=1}{\sum }}H_{i}}\text{.} \end{array}$$

The coefficient of variation method is used as an indicator to show the relative differences between data. This study introduces the coefficient of variation method to study the evolution of regional competitiveness differences in China. Calculated as follows:

(19) $$\begin{array}{c} \begin{array}{c} \delta =\sqrt{\frac{\overset{n}{\underset{i=1}{\sum }}\left(Y_{i}-\bar{Y}\right)}{n}}\text{,}\\ CV=\frac{1}{\bar{Y}}\sqrt{\frac{\overset{n}{\underset{i=1}{\sum }}\left(Y_{i}-\bar{Y}\right)}{\left(n-1\right)}} \end{array}\text{.} \end{array}$$

Here, *δ* represents the standard deviation of the data, CV represents the coefficient of variation, *Y*; represents the data value of the *i* region, and *Y* represents the average value of the entire region.

An important tool for detecting changes in impact is geographic recognition methods. The principle of special calculation is as follows:

(20) $$\begin{array}{c} q=1-\frac{1}{n\delta^{2}}\overset{m}{\underset{a=1}{\sum }}n_{a}\delta^{2}\text{.} \end{array}$$

Here, the value of *q* varies between 0–1. The closer the value of *q* is to zero, the weaker the influence of the influencing factor. The closer the value of *q* is to 1, the stronger the influence of the influencing factor.

### 4.2. Indicator Selection and Data Processing

The collected data is first sorted by year, and the 20 index data contained in the measurement layer of Table 1 are dimensionless and adjusted to eliminate size changes, and the index data becomes a constant value between the merge gaps.

Use the scale conversion method to perform standardization, the formula is

(21) $$\begin{array}{c} y_{i}=\frac{x_{i}}{\max\left(x_{i}\right)}\text{.} \end{array}$$

Here, *Y*_*i*_: the balance value of the indicator; *x*_*i*_: the original data of the indicator; the maximum value (*x*_*i*_): the maximum value of the indicator. Model index system of regional tourism competitiveness development level is shown in Table 1.

According to the entropy method of determining the weight formula:

(22) $$\begin{array}{c} r^{\prime }ij=\frac{\max\left(r_{ij}\right)-r_{ij}}{\max\left(r_{ij}\right)-\min\left(r_{ij}\right)}\text{.} \end{array}$$

Set *b*_*ij*_ = 1 + *r*_*ij*_^ ′ to make 1n*f*_*ij*_ meaningful, and then calculate the entropy value of each index.

*H*_*i*_ is the entropy value of the *i* indicator:

(23) $$\begin{array}{c} H_{i}=-k\overset{n}{\underset{j=1}{\sum }}f_{ij}\times \ln f_{ij}\text{,}\\ \begin{array}{c} f_{ij}=\frac{b_{ij}}{\overset{n}{\underset{j=1}{\sum }}b_{ij}},\\ k=\frac{1}{\ln\;n}. \end{array} \end{array}$$

Here, (assuming *f*_*ij*_ = 0, In*f*_*ij*_ = 0), then the weight of index *i* is obtained:

(24) $$\begin{array}{c} W_{i}=\frac{1-H_{i}}{m-\overset{m}{\underset{i=1}{\sum }}H_{i}}\text{.} \end{array}$$

*W* = *w*1 *w*2 *w*3...]*T*,*W* is the required eigenvector.

### 4.3. Analysis of China's Regional Tourism Competitiveness

**Figure 6:**
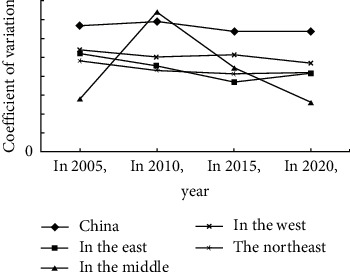
Examines the differences in the level of tourism competition in the four economic zones.

According to the coefficient of variation method, the coefficient of variation of the internal tourism competitiveness of the four major economies in the east, central, west, and northeast was calculated and displayed in a line chart, as shown in Figure 6. The overall difference in China's tourism competitiveness is relatively large, and the overall trend is to increase first and then decrease the fluctuation range. The regional differences between the central, western and northeastern regions are shrinking, and the differences in tourism competitiveness in the eastern region have mainly increased from 2008 to 2010.

The difference in the competitiveness of the tourism industry in the eastern region shows a trend of shrinking and increasing. The difference in tourism competitiveness varies from small to large. The maximum coefficient of variation in 2000 was 0.5244, and the minimum coefficient of variation in 2010 was 0.3641. The difference in tourism competitiveness in the central region showed a trend of increasing and then decreasing. When there was a big change in 2005, the difference in tourism competitiveness in the central region was the largest, and then gradually decreased. The value of the coefficient of variation in 2005 was 0.7495, and the minimum value in 2015 was 0.2513. The difference in the competitiveness of tourism in the western region was between 2000 and 2015. This difference first narrowed and then tended to increase. The difference in tourism competitiveness within the region has hardly changed, and the regional difference is small every year. The maximum value of the coefficient of variation in 2000 was 0.5234, and the minimum value in 2015 was 0.5525. The difference in the competitiveness of the tourism industry in the Northeast region has generally shown a downward trend, and the difference in the competitiveness of the tourism industry in the region has changed significantly, with slight changes in 2015. The coefficient reached its maximum value of 0.4846 in 2000 and its minimum value of 0.4191 in 2005.

Multiply the weights obtained in Table 1 by the standardized index and add them to get the tourism competitiveness level score of each local government department, as shown in Table 2.

The region with the highest tourism competitiveness is in the east. The tourism competitiveness of the central region is lower than that of the east, and the tourism competitiveness of the northeast and western regions is not ideal.

### 4.4. Identification of the Dominant Factors in the Evolution of Regional Tourism Competitiveness Differences

Compared with 2010, the 19 factors affecting the competitiveness of the region have been reduced to 12.5 A-level scenic spots; passenger attrition rate, total tourism income, average staying time, per capita daily consumption, the number of beds in medical institutions, and the number of hotel employees do not have an important impact. These include local fiscal revenue, hotel operating profit, total number of tourists, number of travel agencies, total tourism revenue, and disposable income of residents. The impact on the competitiveness of the region is weak. Per capita disposable income, higher education institutions, road network density, passenger turnover, and the increase in the number of private cars and star-rated hotels in the region have affected tourism competitiveness as shown in Table 3.

According to the analysis of the variance table of the model used in 2000, 2005, 2010, and 2015, see Table 4.

Perform the F test of the regression model and check the confidence level *α* = 0.05 and the F distribution table, you can see that F0.05(20,10) = 2.774 *F* > 1970 > 2.774. In the table, the annual regression model is selected as the model with the highest F value in the stepwise regression. Since 1970, the F values of the four selected models have shown that they have a regression effect on the four cursor models. The evolution of China's regional competitiveness differences and its influencing factors.

After that, in 2000, 2005, and 2010, the 2015 20 index data were applied to the geographic detector model. The analysis and TT calculation results are shown in Table 5. Number of institutions of higher learning, per capita disposable income in rural areas, operating income of travel agencies, travel agencies, passenger transfers, number of private cars, total number of tourists, and fiscal revenues in the region. The result of the geo-detector analysis is that we can see six factors, such as passenger conversion, total number of tourists, private cars, travel agencies, local fiscal revenues, and travel agencies. Operating income helps improve the competitiveness of the Chinese region, and its influence is greater.

The comprehensive regression analysis based on the cross-priority index selection principle and the calculation result of the geographic detector will determine the total number of tourists, private cars, travel agencies, and local fiscal revenue. Four indicators that affect China's competitiveness in the region dominate.

### 4.5. Suggestions for the Development of Regional Types of Tourism Competitiveness

According to the main characteristics of China's regional tourism competitiveness, four regional development proposals will be put forward with reference to the national tourism development policy and research results related to regional tourism development. Tourists are often attracted by novelties when traveling, and often have travel motives, but the homogeneity of tourism products and the high-speed and transparent information distribution between regions are very serious. The development of tourism resources with regional characteristics is of great significance to improving regional competitiveness. Secondly, we need to reveal the more in-depth local culture and tourism resources. With the improvement of people's living standards, the trend of tourism development is to dig deeper into local culture and transform it into iconic tourism products.

Actively promote tourism, social services, and infrastructure construction in the area. In addition, the existing highway toll stations, bus terminals, railway stations, etc., will be upgraded to facilitate traffic inside and outside the area. Further enhance the tourism competitiveness of areas with weak tourism development infrastructure, grasp the main limiting factors of weak tourism development zones, and encourage potential tourism development zones and high-quality resources between regions. The development of the spatial structure of the tourism industry and the improvement of regional competitiveness are constantly developing in the direction of higher quality. At the same time, areas with low tourism competitiveness are improving their competitiveness, actively expanding investment in the tourism industry, introducing investment companies and attracting high-quality tourism talents, cultivating tourism companies, and introducing unique tourism development models and development methods.

## 5. Conclusion

Based on the commercial characteristics of small packet bursts and the stability of random competition in the Internet of Things communications, this paper provides a stable transmission method based on NOMA, which does not require distributed scheduling. According to the Foster–Lyapunov level, we quickly used a series of methods to study the robustness of the nondistributed scheduling system to provide reliable noncooperative distributed transmission probability control. When designing the human-computer communication system, Qt oscilloscope firstly described the development history and status quo of internal human-computer communication, and combined with the oscilloscope's requirements for the human-computer interaction system, it analyzed the entire framework of the oscilloscope and human-computer interaction system and analyzed and analyzed one by one. Realize the entire design of the human-computer interaction system.

## Figures and Tables

**Table 1:** Model index system of regional tourism competitiveness development level.

Target layer	Criterion layer	Measures layer
The development level of regional tourism competitiveness	Supply competitiveness	The total turnover of tourism in ten thousand yuan) (X1)
		Passenger turnover (100 million person-kilometers) (X2)
		Average length of stay (days) (X3)
		Total number of tourists received (10,000 person-times) (X4)
		Cost per person per day (USD per person day) (X5)
	Guaranteed competitiveness	Number of 5A-level scenic spots (a) (X6)
		Total number of travel agencies (number) (X7)
		Average room occupancy rate (%) (X8)
		Number of civilian passenger vehicles (units) (X9)
		Number of star-rated hotels (units) (X10)
		Number of beds in health institutions (units) (X11)
		Highway network density (km/km2) (X12)
		Number of employees in travel agency (person) (X13)
	Economic competitiveness	Number of employees in star-rated hotels (persons) (X14)
		Number of colleges and universities (offices) (X15)
		Local fiscal revenue (ten thousand yuan) (X16)
		Operating income of travel agencies (thousand yuan) (X17).
		Per capita disposable income of urban residents (yuan) (X18)
		Per capita disposable income of rural residents (yuan) (X19)
		Hotel operating income (thousand yuan) (X20)

**Table 2:** Scores of tourism competitiveness in different years in different regions.

Situation in each region		2000 tourism competitiveness level score	2005 Tourism Competitiveness Level Score	2010 tourism competitiveness level score	2015 tourism competitiveness level score

East area	Shandong	0.216	0.275	0.378	0.256
	Jiangsu	0.271	0.362	0.462	0.302

Central region	Shaanxi	0.096	0.112	0.162	0.116
	Shanxi	0.066	0.113	0.141	0.095

Western region	Inner Mongolia	0.054	0.066	0.113	0.085
	Qinghai	0.146	0.165	0.048	0.183

North-east area	Heilongjiang	0.092	0.103	0.126	0.073
	Jilin	0.066	0.112	0.105	0.075

**Table 3:** Table of stepwise regression coefficients in 2015.

	Unbalance factor B	Standard error	Standard coefficient	T	SIG	Poor statistical report capacity	VIF

(Constant)	0.002	0.005		0.235	0.816		
Restaurant operating income (thousand yuan)	0.112	0.004	0.236	12.423	0.000	0.042	19.162
Total number of tourists received (10,000 person-times)	0.364	0.003	0.632	124.351	0.000	0.861	1.152
Number of employees in travel agency (person)	0.063	0.006	0.151	7.198	0.000	0.053	18.577
Highway network density (km/km2)	0.025	0.003	0.061	6.872	0.000	0.269	3.832
Local fiscal revenue (ten thousand yuan)	0.155	0.002	0.265	50.710	0.000	0.898	1.113
Total turnover of tourism (ten thousand yuan)	0.057	0.004	0.184	14.864	0.000	0.165	5.77
Number of colleges and universities (offices)	0.028	0.005	0.068	5.304	0.000	0.148	6.753
Per capita disposable income of residents (yuan)	0.031	0.004	0.036	4.883	0.000	0.284	3.323
Number of vehicles owned by civilian passengers (units)	0.0364	0.004	0.087	9.341	0.000	0.237	3.920
Per capita disposable income of urban residents (yuan)	0.030	0.004	0.047	7.842	0.000	0.689	1.452
Passenger turnover (100 million person-kilometers)	0.032	0.004	0.046	5.657	0.000	0.241	4.232
Number of star-rated hotels (units)	0.015	0.004	0.035	3.334	0.004	0.224	4.457

**Table 4:** Variance table.

Model	Sum of square	df	Mean square	*F*	Sig.

2000 regression model	0.244	10	0.024	1970.915	0.000n
2005 regression model	0.449	19	0.024	4215326.708	0.000u
2010 regression model	0.577	19	0.03	8879209.509	0.000u
2015 regression model	0.311	12	0.026	3402.186	0.000n

**Table 5:** The q value of each indicator in the four years.

Index	2000	2005	2010	2015
Total tourism revenue (ten thousand yuan)	0.35	0.38	0.45	0.43
Passenger turnover (100 million person-kilometers)	0.52	0.49	0.47	0.51
Average length of stay (days)	0.19	0.23	0.27	0.29
Total number of tourists received (10,000 person-times)	0.61	0.51	0.60	0.67
Average daily cost per person (USD/person Yao)	0.29	0.32	0.37	0.34
Number of 5A-level scenic spots	0.36	0.41	0.49	0.43
Total number of travel agencies (a)	0.39	0.41	0.53	0.45
Average room occupancy rate (%)	0.25	0.23	0.37	0.31
Number of vehicles owned by civilian passengers (units)	0.56	0.57	0.64	0.35
Number of star-rated hotels (units)	0.32	0.35	0.51	0.49
Number of beds in health institutions (units)	0.37	0.31	0.28	0.32
Highway network density (km/km^2^)	0.35	0.31	0.28	0.34
Number of employees in travel agency (person)	0.47	0.49	0.43	0.51
Number of employees in star-rated hotels (persons)	0.27	0.35	0.41	0.39
Number of colleges and universities (offices)	0.39	0.37	0.41	0.38
Local fiscal revenue (ten thousand yuan)	0.57	0.61	0.68	0.63
Total turnover of travel agency (thousand yuan)	0.41	0.57	0.60	0.54
Per capita disposable income of urban residents (yuan)	0.36	0.34	0.32	0.35
Per capita income of rural residents (yuan) (X19)	0.38	0.47	0.42	0.40
Hotel operating income (thousand yuan)	0.19	0.21	0.23	0.26

## Data Availability

The data used to support the findings of this study are available from the corresponding author upon request.
